# Exploring the experience of children with disabilities at school settings in Vietnam context

**DOI:** 10.1186/2193-1801-3-103

**Published:** 2014-02-20

**Authors:** Kham V Tran

**Affiliations:** University of Social Sciences and Humanities, Vietnam National University, Hanoi, Vietnam

**Keywords:** Children with disabilities, Vietnam, Social inclusion, Inclusive education

## Abstract

The initial findings from 230 questionnaires’ survey and 36 interviews, in which informants are CWD, children with non-disabilities (CWND), parents of CWD, and teachers in school settings, are stated as: (a) the general understanding of disability is based on medical model and individual model rather than social model, such understandings contribute great impacts to the CWD’s experiences in their daily life in general and in school contexts in particular; (b) the most important difficulties which CWD experience at school are those of learning facilities, the empathy from their student peers and barriers in the physical environment; (c) the ways which CWD try to deal with such difficulties are mostly ‘do-by-themselves’ or try to adapt themselves rather than asking for supports actively. Based on these findings, recommendations for having further activities to change social awareness of disabilities, specific support structures for CWD and school staff are stated in order to promote the social inclusion of CWD in schools.

## Introduction

Recent reports by Vietnam Ministry of Education and Training (MOET) and Vietnam Ministry of Labour, Invalid Soldiers and Social Affairs (MOLISA) stated that just only nearly one-third of children with disabilities (CWD) were going to schools (MOLISA 2004 MOET 2010). Meanwhile the rest are unable to go to schools and as its consequences, their social inclusion is partial. Even those in school settings are facing and experiencing many difficulties daily in inclusive education or in special education. Such difficulties range from negative social attitudes towards disability and CWD, to lack of learning facilities and shortage of skilled teachers and support staff (MOET 2010). This paper, as a main part of my thesis on social inclusion of children with disabilities in Vietnam, focuses at examining these difficulties in the Vietnamese context and makes suggestions for promoting social inclusion of CWD in the schools in the Vietnamese social, cultural and political contexts.

The research process by Crotty (Crotty 1998) is applied in the research in which social constructionism, symbolic interactionism and ethnomethodology are used as the theoretical background and approach for the research. The main methods for collecting data in terms of observation, survey and interview are followed by such approach. Research questions stated in this paper are in terms of how CWD experience their difficulties in schools? And how they can deal with such difficulties?

The initial findings from 230 questionnaires’ survey and 36 interviews, in which informants are CWD, children with non-disabilities (CWND), parents of CWD, and teachers in school settings, are stated as: (a) the general understanding of disability is based on medical model and individual model rather than social model, such understandings contribute great impacts to the CWD’s experiences in their daily life in general and in school contexts in particular; (b) the most important difficulties which CWD experience at school are those of learning facilities, the empathy from their student peers and barriers in the physical environment; (c) the ways which CWD try to deal with such difficulties are mostly ‘do-by-themselves’ or try to adapt themselves rather than asking for supports actively. Based on these findings, recommendations for having further activities to change social awareness of disabilities, specific support structures for CWD and school staff are stated in order to promote the social inclusion of CWD in schools.

### Research methods

Methods for collecting and generating data and implications for satisfying the research aims in this paper, based on the outcomes of the research on “Social inclusion of children with disabilities in Vietnam”, consist of interview and survey as the main methods of data collection on the research process, based on the model of Crotty, which includes four significant elements as: Epistemology, theoretical perspective, methodology and research methods (Crotty 1998).

#### Survey

Survey’s research populations are included as: CWD, CWND in inclusive schools, parents of CWD, teachers and community persons who experience their life with CWD. The questionnaires are delivered in school and families with CWD and to those people living around CWD’s houses. In order to make the simplicity of survey data, research participants are grouped into PWD and PWND.

There are three parts on the survey. The first part consists of 7 questions on general information. The second part includes 3 main questions in terms of knowledge, awareness and practice toward disability. And the third one has 5 questions on daily activities experienced by CWD. Research participants, including CWD, CWND, teachers, parents of children with/without disabilities, are chosen in the inclusive schools in one district of Hanoi, Vietnam. They are free to attend this research. This research focuses only CWD in types of mobility and vision impairment. For those CWD in term of visionary, the researcher reads aloud the content of survey and write-down the answers. The total number of research participants attended is 210. In which 9.1% of respondentsis PWD,. Among respondents, the male counts for 32.4%. At the category of education level, there is 31% for primary level, and 7.1%, 4.8% for secondary and high school levels respectively while the rest rate, around 57%, is at college and post college levels. About the career, nearly a half of respondents are student at all levels, and the teachers in this category count for 22.4%. The youngest is at 10 years old, as at the 4th grade in Vietnam education system, while the oldest is around 65 years old. The age group of those under 18 years-old, recognised as a child group in Vietnamese regulation, is 39.3% which higher than that rate in Vietnamese population (35.2%) (General Statistic Office (GSO) 2010). The quantitative data is generated by application of SPSS software to have additional statistical values for data explainations in details.

#### Interviews

Interviewing is a useful and significant tool in social research through in-depth interviewing and focus groups. The content of the interview and focus group focuses deeply on the aspects of daily activities of CWD, such as how to make friends in school, how to experience the difficulties in school, in family and in community. These methodd aim at collecting the qualitative data. Each interview is about a half hour to an hour in duration and audio recorded. Note taking during interviewing was used as the reference for the content of interview. All interviews were in Vietnamese and done in contexts of family or of school. There are 34 interviews from CWD, parents, teachers, CWND, neighbors and community leaders.

These methods are followed by the guidelines with ethical approvals by University of South Australia (2009), number P140/09. Written informed consent was obtained from CWD’s parents, teachers for publication of the research report.

## Findings

### Disability situation in Vietnam

#### Vietnam on its development process

Vietnam is located in South East Asia with 329,560 sq. km of its surface and 89 million people in 2010 (UNDP 2010). In the Declaration of the independence of Vietnam in 1945, while the country was faced with varieties of enemies including famine, ignorance and foreign invaders, President Ho Chi Minh solemnly stated that the rights to live and pursue happiness were significant, fundamental, supreme and inalienable rights for every individual and every nation. These rights were also Vietnam goals in all national actions and plans for entire Vietnamese (Minh 1945; Nam et al. 2001). The implications of this Declaration were consistent with national humanitarian tradition throughout its thousands year of history. And they were also combined between permanent human values and Vietnam cultural tradition: *Development for people, by people and of people* (Nam et al. 2001).

Before 1986, central planning was the government administrative mechanism; all activities of economic and social life were conducted by stated-owned enterprises or cooperatives. During this period, all social welfare and activities toward PWD were operated by only financial support from government. The subjects of such welfare were limited.

In 1986, the Vietnam Government began to implement the “Doi Moi” reforms in which the central planning was replaced with market orientation. In its results, there were three significant changes including (a) de-collectivisation, (b) land processing for household and (c) trade liberalization. After two and half decades of implementing “Doi Moi” policy, it is witnessed the rapid growth in economic and social life. The GDP has been growth sustainably.

Turning to the 21st century, Vietnam made great steps on integration with regional and international countries. Vietnam became the 150th member of WTO since 1st January 2007. Vietnam has achieved rapid economic success and remarkable social progress, reaching lower middle income status in 2009 as well as the leading country in the Asia-Pacific region on achieving the aims of the Millennium Development Goals (MDGs) (2010).

The first decades of the 21st century, Vietnam identified its development strategy in the title of “Economic and Social Development Strategy for the period of 2001-2010”. On evaluating this Strategy, Vietnam Government confirmed that the GDP for this period increased with 7.2% annually. The GDP per se is also increased 4 times by the end of this period. Such achievements also created the great changes of social life as well as social infrastructure. Vietnam recorded kept the critical achievements in all aspects: Political stability, sustainable economic growth, extensive democracy in all corners of social life, poverty reduction in line with social equality, and ensured public social security (Vietnam Government 2010).

Its economy got relatively rapid and sustainable growth with a dramatically and sustainably increased GDP for last decades, in spite of the chaos in the world economic. The GDP per capital is still at low level however it increased sustainably. With its economic development, the investments on education, health care, social welfare and social services have been focused with priorities in recent years, which contribute to the upgrade of human development index (HDI). Vietnam got its HDI rank of the 113th in the world (UNDP 2010).

In promoting social development, Vietnam set up the new strategy for the next decade of 2010s. The strategy identifies the main aim of: “By the end of 2010, Vietnam will be the industrialised country. In which the social life will be stable, democractic and legal. In additions, there will be progress on mental and material life for all; consistency on the national unification and sovereignty; highly progress of social status of Vietnam in the world and making significant contribution and background for the next developing period”. (Vietnam Government 2010).

In summary, during the time of reluctancies in all aspect of social life worldwide, with the internal efforts by all agencies at different levels and of the people, Vietnam keeps its development direction sustainably. The income of the population increases year by year, living standards of the whole population has remained stable. As it consequencies, social policies for poor regions, poor districts and poor people, income and living standards of rural areas, remote areas, poor area and poor people have been stablised and improved critically (GSO 2006).

#### Overview of PWD in Vietnam

In Vietnam, there is not a national and comprehensive survey on disability that states the reliable estimates of the overall statistics on disability and its types as well as its variations on age, gender, social status and others characteristics (Bao 2001). So it is lack of concrete and exact rate of PWD in Vietnam. Recently, various organisations in Vietnam conducted some disability survey. However, they were often small scales and the collected data is used for specific purposes or intentions on implementing functions and tasks of ministries areas (Bao 2001). At the paper on overview of disability in Vietnam, Dr Bao identified that the concept, definition of disabilities, classification of types of disabilities, variables, indicators, content and methodologies used by various agencies in the previous surveys were inconsistent and unclear which led to the underreporting or over-reporting of disability data (Bao 2001). Almost research on disability in Vietnam in last few years cited the statement of disability rate from MOLISA’s annual reports.

Up to now, there has been not exactly rate of PWD. The survey on living standards in 2006 showed this rate, accounted for PWD from 5 years old was 15.3% in which its rate was 17.8% in urban and 14.4% in rural. The age group of 5 to 17 accounted for 13% (GSO 2006, p.167). This survey also identified the disability in 6 types of visual, hearing, intellectual, mobility, communication and self-caring. In other approach, the National Census on 2009 found out the rate of PWD, above 5 years old, in Vietnam was 7.8%, in which 53.8% was female group and 75% of PWD lived in rural areas (GSO 2010). MOLISA estimated a total of PWD in Vietnam of 6.3% (approximately 5.3 million people), which also led to nearly 8% of Vietnamese households, included a PWD and most of them are in poor social and economic condition. Of them, 1.5 million were classified as “heavily disabled” (MOLISA 2004; Bao 2001; Duong et al. 2008). This rate, in referred from the term of WHO indicates, should be 10% of the total population.

Basing on the annual reports on promoting rights for PWD by the United Nations Economic and Social Commission for Asia and the Pacific (ESCAP), it is found that Vietnam has its high rate on PWD among its demography, around 6.4%, in composition with other countries in the region. In details, the proportion of males with disability was higher than that of females, 63.5% in compared with 36.5%. About 16% of PWD is in age group of under 16 years old while that of group from 16–55 is 61% and that of group above 55 year were 61% and 24% respectively (Duong et al. 2008). The major disabilities include mobility (29.4%), mental (16.8%), hearing/speaking (16.4%), and visual disabilities (13.8%). Also, up to 20% of the PWD are multi- disabled. This statistic is quite different from the 2009 findings (GSO 2010). The main reason for the different in the statistics on PWD in Vietnam as there is lack of concrete definition on disability and there is different ways on data collection of disability situation recently.

On looking at the causes of disability, over one third of disability was congenital. Another one third was caused by disease. It is significant to note that war-related causes explained the disability of one quarter of PWD, according to the government’s own figures. It is estimated that the proportion of PWD in the total population will increase in years to come due to traffic or work accidents, and environmental pollution brought about by rapid industrialization and urbanization (Duong et al. 2008; The United States Agency for International Development 2005; MOLISA 2004).

#### Overview the life of CWD

Children in Vietnam, under 18 years old, accounts nearly 31% of its population (26.2 million) which comprises the significant part of Vietnam population (General Statistic Office 2010). As the general statistics on PWD in Vietnam, there has been not any official rate about the number of CWD national wide. Almost these numbers are based on MOLISA, MOET or MOH annual reports. Among children population, it is estimated that about 662 thousand are disabled accounts for 2.4% of its population group (MOLISA 2004; UNICEF Vietnam 2010).

It is recognised that, as other child, a child with a disability, in all types, has the potential to grow within his or her community and to affect their lives of people living around.

Among CWD, the severe type counts for 31%, the others including types of hearing, visionary, mentality, language, mobility and others are 15%, 12%, 27%, 19%, 20% and 7% respectively. Especially those children of learning difficulty account the highest rate with 28.36% (MOET 2010b; MOLISA 2004).

In the report about the inclusive education for CWD by MOET in 2010, it identified the causes for being disabled in which children are disabled by congenital with 72.38%, by illness with 24.34%, accident with 3.93% and at born with 2.28%. This report also provided the overview of the life of CWD. It is stated that 55.67% of CWD living in low income families, 41.41% of those CWD living in middle income families and that rate in above middle income families counts for just only 2.92%. Especially, it is about 61.38% of children with multi-disabilities in families with economic difficulties. So, there is limitation of rate on CWD going to school. CWD attend mostly in some convenient institutions (such as the center for impaired people by the Blind Association at the district levels and organistions by disabled people) and special education centres (MOLISA 2004; MOET 2010b).

Vietnam, as a developing country, has been trying its best on creating welfare states and delivery the comprehensive social policies on making the harmonised society with equalities. In spite of rapid progression recently, as much of the world, CWD face difficulties in accessing their physical environment, accessing to the community-based services and health care, education, and child protective systems.

In pursuing with dealing these limitations, the Vietnam Government signed the international conventions particularly on rights of child (1986) and rights of people with disabilities (2006), which made signals that Vietnam is committed to adapt society to meet the needs of CWD.

There is lack of comprehensive research on disabilities as well as the life of CWD in Vietnam. Almost recent assessments based on the national survey in 1998 by UNICEF; research on children with disabilities in 2004 by MOLISA and annual reports by MOLISA, Provincial Depaprtment of Labors, Invalids and Social Affairs (DOLISA). As the contents of these research, life of CWD is viewed in terms of accessibility, community based services and health care; education, institutionalisation; child protection system; citizen involvement.

### Social inclusion

#### Values of inclusion and inclusive education for CWD

Responds on these sub-questions are grouped into 3 options: Not agree, No ideas and Agree. The statements are about the general understandings about inclusion in terms of benefits for CWD and PWND in relations with CWD as in Table 1. The formers include the benefits in forms of developing skills on socialisation, self-image, sensitivity, learning and language development, having good conditions for later life participation and have more chances for making friends, while the latter consists of those about the meaning of inclusion for both CWD and CWND as well as more understanding CWD.

**Table 1:** **Knowledge on social inclusion**

No	Knowledge on social inclusion	Not agree	No idea	Agree
1	CWD could learn socialisation skills from CWND	2.9	7.6	89.5
2	CWND would learn to develop sensitivity to CWD by having opportunity to know CWD	2.4	9	88.6
3	Teacher would learn to promote sensitivity to CWD by having chance to know CWD	3.8	8.6	87.6
4	CWD could learn cognitive and language skills from CWND	3.8	9.5	86.7
5	Parents of CWD believe integration/social inclusion is best for their children	3.3	11.4	85.2
6	Parents of CWND believe integration/social inclusion is best for their children	7.1	15.7	77.1
7	CWD would develop positive self-image	7.6	15.7	76.7
8	CWD would be well-prepared for later participation in regular education and in society	8.1	26.2	65.7
9	CWD would have more opportunities to have friends	23.3	28.6	48.1

Values on social inclusion have been acknowledged for both CWD and CWND in terms of socialization, developing sensitivity as well as for later social integration for CWD. Parents of both CWD and CWND also get benefits on social recognitions for their kids. However, idea on “CWD would have more opportunities to have friends” is responded with low rate.

In transcribing interviews as well as from observation, this situation seem consistency due to the type of inclusion in Vietnam now is still in aspect of locational integration (i.e., it is less social integration, only creating conditions for CWD to go to school, and lack of social assistance to CWD as well as CWND) rather than social inclusion.

By looking at these statements’ means statistically, all these means are closed to value 4, with meaning that agreed, while only last statement is at 3, meaning of “no ideas”. Value of these means expressed the implication that the responds are quite positive on the general understanding of social inclusion. In additions, the means about statements on values for CWD are less responded than those values for PWND. Understanding social inclusion is explained through the experiences of CWD and CWND as well as the meanings for not only CWD but also for PWND including their parents, CWND, parents of CWND and teachers in working with CWD. Almost ideas express the meaning for CWD in terms of making friends, relationship and getting opportunities for further learning and inclusion. While ideas on encouraging CWND and their parents on deeply sympathy with CWD are also expressed, these situations create the significant background for increasing social awareness and supports towards PWD.

#### Social inclusion: implications from daily activities

In this section, social inclusion is investigated in the ideas of daily activities in which CWD participate with CWND as in Table 2.

**Table 2:** **Statements on daily activities of CWD and CWND (%)**

	Never	Rarely	Sometimes	Frequently	Always
CWD play with other CWD	3.3	5.2	29	41.5	21
CWND play with other CWND	4.8	5.7	21.9	20.5	47.1
CWND help CWD	3.8	10	33.3	28.1	24.8
CWD play with CWND	3.8	14.8	49.5	25.2	6.7
CWND play with CWD	3.3	15.7	48.1	22.4	10.5
CWD initiate interaction with CWD	8.1	12.8	28.6	33.8	16.7
CWND initiate interaction with CWND	6.7	14.3	22.4	29.5	27.1
CWND prefer to play with others than CWD	10	14.8	31	29.4	14.8
CWD prefer to play with CWD than CWND	11	18.1	28.1	25.7	17.1

Responds are expressed with 5 options in Likert’s scale which are: Never {1}; rarely {2}; Sometime {3}, frequently {4} and Always {5).

The most significant activities, which are frequently initiated, are “CWND play with CWND” and “CWD play with CWD”. These statements got higher rates than those statements on “CWND play with CWD” and “CWD play with CWND”. This situation is meant that the separated groups of CWD and CWD are still existed in daily activities involving CWD and CWND.

### Identify the difficulties of CWD at school

In the list of coding the conversations, it is found that “the difficulty at school”, in general, is at high ranking around codes taken from all interviews, observations and fieldwork notes which demonstrates the most ideas from interviewees, PWD and PWND referring to the most difficulties in school.

The difficulties are encoded freely in all aspects of CWD in schools, and then they are grouped into categories of learning and its facilities, moving and social attitudes. The findings achieved from interviews with CWD, teachers, parents and CWND in schools.

Quite different with many research findings (Duy 1995; Japan International Cooperation Agency 2002; Duong et al. 2008; van Kham et al. 2005) on looking at the difficulties expression by CWD which are prominent in interview and information from CWD, the interviewees, children with disability, in this research express the difficulties simply from educational facilities, the limitation of physical environment more rather than social attitudes from other children, as well as from society.

The ways to get information for CWD are through interview and participation observation. As mentioned in the method section, this research applied ethnomethodology, so the researcher paid more time in the field before starting collecting data.

The main question asked for collecting data is around the idea of “Would you please tell me your difficulty/difficulties at school?” and its sub-ideas developed suitably in each interview.

#### Barriers and difficulties in schools in term of learning

In this section, questions for CWD are raised around the content of “what is your most difficulty in learning?”, and those for teachers are around “what is your most teaching difficulty in inclusive classroom?” These questions are directly or indirectly asked in almost interviews with these informants.

- Teachers

Less experienced of teacher in area of inclusive education is acknowledge as the first and prominent difficulty for CWD in schools. Almost teachers in inclusive institutions are lack of knowledge and skills on teaching and working with CWD as well as CWND in order to promote inclusion. This situation is due to the limitation on pedagogic system in which almost programs exclude training teaching students with knowledge and skills on teaching in inclusive settings.

On looking at almost curriculums at training teachers for primary, secondary and high schools from programs in Hanoi University of Education and Hanoi College of Education, it is found that except the Undergraduate Program on Special education, there is not any specific subjects on inclusive education or teaching the children in special need (Cao đẳng Sư phạm Hà Nội 2011; đại học Sư phạm Hà Nội 2011). There are only some related subjects which include implications on the subject’s contents about children with disabilities or teaching with special need children, such as on Psychology of Aging, Children Psychology, Educational studies, Theories on teaching at primary level or Outdoor activities. So, almost teachers are lack of professional knowledge and skill on working with children in special needs in general and CWD in particular. Only program in Special Educations, a range of subjects on inclusive education and teaching with specific type of CWD has been included. Recently, Ministry of Education and Training delivered the training program on inclusive education for training teachers in university, potential teachers and staff in educational institutions. This program is applied in pedagogic universities and colleges with expectations on providing knowledge and skills on inclusive education for training teachers at all levels (MOET 2010a).

Teacher is acknowledged as the important factor for social inclusion in school, especially the image of teacher is really essential from children’s perspective. In Vietnam cultural values, the symbol of teacher is very important from not only the view of children (learners) but also the view of children’s parents, there are some proverbs as “không thầy đố mày làm nên/ You are unsuccessful without the masters” or “Muốn sang phải bắc cầu kiều, muốn con hay chữ phải yêu lầy thầy/ if you want to cross the river, you must build bridge; if you want to be good, you must follow teacher”. So, the impacts from teachers play an important role for children’s outcomes in academic and non-academic areas significantly.

In survey, when talking about the role of teacher on promoting social inclusion for CWD, 86.7% of respondents refer such role is important.

“… Teachers are unable to understand the Braille” (DBT, male, 12 years old, visual disability).

“… Teachers in my home town school do not know the ways to teach CWD, to read the Bray, so the way to make the examination is quite complicated” (LM, male, 14 years old, visual disability).

“… There is not any high school for accepting children with visual disability” (LBH, female,13 years old, visual and physical disability).

“… Teacher does not help anymore” and “there is not any help from teacher, just only place me on the first rows in classroom” (THD, male, 15 years old, visual disability).

- Educational facilities

The second difficulty that is stated by CWD is about the educational facilities. It is found that there are less educational facilities, as shortage of textbooks and other supplementary for CWD on inclusive education.

For those in visionary difficulty, the shortage of textbooks and learning supports for studying is an important factor for their learning.

On talking social policies on inclusive education: NVT (CWD) identified the weakness of inclusive education policies on encouraging PWD go to school previously:

“… At that time, if I want, it is impossible, in fact there is not any guideline, now it is still consistency excluding the special schools for CWD” (NVT, male, 16 years old, visual disability).

- “… I found that they do not pay enough attentions to inclusive education and the teachers have not been trained with skills on working with CWD, so they do not know the suitable ways on teaching us” (NVT, male, 16 years old, visual disability).

“… The most difficulty is about book and material for learning” (DBT, male, 12 years old, visual disability).

“… There is difficulty in lessons while learning in here, meanwhile that difficulty is in book and other learning material. The lessons become more and more difficult, so I feel to be more difficulty” (DBT, male, 12 years old, visual disability).

“… CWD is unable to see whatever in the backboard” (DTH, female, 14 years olde, visual disability).

“… Just listening to the content of teacher’s lesson” (LBH, female,13 years old, visual and physical disability).

“… There are not any practice books, just only the text books” (LBH, female,13 years old, visual and physical disability).

“… In my home village school, there is lack of learning materials, almost CWD write themselves in order to have the reference text for learning” (LM, male, 14 years old, visual and physical disability).

Talking about learning at school:

“At home, I also learn the next lessons previously without any assistance so it is slow progressive, the best way is to exert myself” (LM, male, 14 years old, visual and physical disability).

“The most difficulty in my learning is the lack of textbooks” (LHH, female, 14 years old, visual disability).

“… In learning, the most difficulty is after absent from school due to illness, when I come back it is difficult to understand the lessons” (LHH, female, 14 years old, visual disability).

On talking about material for studying:

“Besides the textbooks, we have not got any other learning materials” (NVT, male, 16 years old, visual disability).

“… The most difficulty is that I am not able to see what teachers write down in blackboard, I must ask for help from other kids, some time they are unable to write down so they do not read aloud for me” (TVB, male, 14 years old, visual disability).

“… The most difficulty is how to understand the lessons” (TVB, male, 14 years old, visual disability).

“… The textbooks for CWD are not available as those for CWND, CWND normally write down and do the exercises, I have not got any textbooks so I need assistance from other CWND” (TXT, male, 14 years old, visual disability).

“About learning, the textbooks are not enough. For example, in the Math’s subject, I want to have another reference book but it is not in the Bray, so I need other read aloud, it is sometime inconvenient. I meet many difficulties in learning. As you know, doing exercises takes only few minutes for CWND, but it takes me for longer due to the touching of the Bray. I like Maths but the number written down is not easy for reading, meanwhile doing Maths exercise must follow step by step. CWND can see what written, but I can’t see anything even it is written in blackboard. Reviewing takes me a lot of time. CWD like me must remember lessons, it is quite difficult at first, it is hard to remember all at once” (VN, female, 15 years old, visual disability).

On talking the difficulties of CWD, voices of CWND are concerned to some aspects of walking, learning and following activities in schools. Responses from 6 CWND in schools also express the difficulties of CWD in learning materials and the ways on achieving the contents of lessons.

“… They found difficulties on walking, doing exercises as well as making contribution during class in order to get the encourage mark in each term” (NNY, female, 15 years old, CWND).

In this area, knowledge and skills on teaching in inclusive education is very important, in additions materials for teaching CWD in specific type of disabilities (hearing and vision disabilities) also contributes great impacts to the learning process. For those CWD in term of physical one, it is rarely to get their voice on talking about the difficulties on learning materials and facilities.

#### Barriers and difficulties in schools in term of mobility and physical environment

The accessible way or pathway for PWD is neglected in almost streets; public places and transportation, in spite of the requirements of having ways and facilities for PWD in new buildings have been approved in legislation and fundamental social policies for nearly 10 years. Almost schools in Hanoi, were built previously or recently, are less accessible for PWD, especially those are special schools. That is one of limitations for PWD to access and involve in activities with peer students in schools.

In the interviews, the some questions and sub-questions are raised about: *How you go to school, with yourself or other assistance? How you find difficulties at schools? How you find the restrictions when you attend out-school activities?*

Almost voices of CWD expose the difficulties on moving and walking in the inaccessible physical conditions. These conditions are about the way for wheelchair, the stair steps, limited playground, and places for outdoor activities. The following extracts are taken from interviews with CWD (visionary impaired):

“… There are stairs in walking ways, so I sometimes strip over steps” (NDT, male, 15 years old, visual disability).

“… I found that there are restrictions in moving in playgrounds and the walking ways” (NDT, male, 15 years old, visual disability).

“… When I participated the outdoor activities, I found that it is very difficult due to I am not familiar to the ways. It is very hard to attend.” (NQH, male, 11 years old, visual disability).

“… I think that these stairs should be slope gently, which will make us easy on moving” (NQH, male, 11 years old, visual disability).

“…Oh, at first, I found that this school is quite big… it is difficulty on moving, but I gradually make acquaintance with such and everything becomes normal” (DBT, male, 15 years old, visual disability).

“… Yes, when I started here, I am very worried about the environment here, because I am unaccustomed and remembered the given room” (LHH, female, 14 years old, visual disability).

“… It is said to be or not to be suitable, all are not reality. Because, the school was built for a long time, so it is not suitable with present requirements. Some places are really insecurity, such as the square pillars, someone had hit their head into them” (LHH, female, 14 years old, visual disability).

Observing at 5 schools in my research, there is same problem of inaccessible environments for PWD moving and attending outdoor activities. There are also no accessible toilets. All the ways and pathways are inaccessible. This situation is consistent in almost schools in Hanoi as well as in Vietnam.

From interviews, having assistance from other children, but normally on specific activities for studying, is preferable and popular, it is hard to get any clues about the assistances in areas of mobility as well for further social inclusion. And in some sections of interviews in school settings, it is found that almost CWD learnt in higher floors with inaccessible pathway and stairs, so at the breaks between class time, CWD stay inside class while other CWND go out for playing. Further, at the age of primary and secondary level, children are lack of concentrating on supporting other while they are not taken any instructions or warning from the adult or teachers. In schools, teachers always divide the responsibilities on learning supports for CWD from CWND, so the supports in learning are clearer in school settings than other tasks and activities.

In spite of the difficulties faced by the physical environments, many CWD find the best way for deal with them by self-adaptation than make the negative voice to that situation. When talking about the expectation for changing the life condition, the opinions on physical environment are less acknowledged.

Basing on the ideas on social constructionism, the way CWD create the meaning in living, in having social inclusion in their setting are socially constructed basing on their reality and focusing on how to maintain their present status than requiring more critical conditions. In this research, CWD always construct their meanings of their happiness and luckiness on going to school, so experiencing the foreseen difficulties at school is also better than those did not have a chance to go to school like them.

#### Barriers and difficulties in schools in term of social attitudes

In schools, some CWD also experienced the bad attitudes from other kids as well as from teachers on looking at their social position, abilities in learning and playing as well as prospects of CWD. From CWD’s experiences, they find more difficulties in term of social attitudes in inclusive environment rather than in semi-special school, as in Nguyen Dinh Chieu Schools. That experience is also existed in interaction with teachers. There are some forms of bad social attitudes: Not paying attention, annoying, not sympathetic, negative labelling, insulting, discrimination. To sit down under other kid’s bad behaviours is also the response from CWD in inclusive schools.

“… In fact, I meet some kinds of bad social attitude. People are different. Not all of them pay good attention to me” (DBT, male, 12 years old, visual disability).

“… The most difficulty I found in learning with other CWND is such the compassionate from them. It is lack (NQH, male, 11 years old, visual disability).

“… CWND do not pay attention to us, they just read aloud the content of lessons only” (TVB, male, 14 years old, visual disability).

“… Oh my god, they annoy us a lot, sometime they did intend to hit us after school hours (TVB, male, 14 years old, visual disability).

“… There are many kinds of attitudes on discrimination in QM School, such as two other girls in front table always told me as crazy or mad man… I must accept that because it is inclusive environment” (TVB, male, 14 years old, visual disability).

“… In my home town school, there are 10 teachers, but not all of them want to understand my situation, as well as the difficulties in my life, there is also discrimination in teacher attitudes… for example, teachers do their tasks for the school’s responsibility only…. (TXT, male, 14 years old, visual disability)

“… I am very sad to see that only teacher in English and the principal pay attention to me. The senior master just only considers to our class issues without any concerns with me even I don’t require any further assistance to my life situation, he does as his responsibility. (TXT, male, 14 years old, visual disability).

The social attitude is being progressive after CWD had a chance to play, learn and go with CWND. Many ideas expressed that it is recommended to start inclusive education from early year, as from preschool activities as well as having more chance for children to play together inside and outside school settings.

“… At first, the CWND do not understand us, they always annoy us, afterward they change their attitude, they have stopped tease us” (LTT, female, 16 years old, visual disability).

As explained in findings from survey, at school in spite of CWND express their willing and feelings on supporting CWD that is required from teachers, CWND as well as CWD aim at playing together in their groups rather than in mixed groups. Negative attitudes seem to be stated more clearly in those schools CWD participated in later class (i.e. not attending from their first years) as well as in those schools with lacking in inclusive teachers and inclusive materials and facilities (in almost schools of CWD’s hometowns).

*In brief,* CWD face difficulties in school settings daily, which are ranged from social attitudes to physical environments and learning facilities, it leads to low self-imaged by CWD. Some expressions by CWD focused on their worries about how to follow up learning at high school level and higher. These existed understandings by CWD are originated from their older friends and the limitations on higher education for PWD, which they got in media and their social networks.

## Discussions

### Barriers and understandings of disability

In this research, some parts of survey and interviews focused on the social construction of disability, which is aimed at providing the knowledge on this aspect in specific contexts. As mentioned, in Vietnam, the term of disability (khuyết tật) has become popular in academic papers and documents recently after Vietnam signed the United Nations’ Convention on the rights of disabled persons in 2006 and approved Law on PWD in 2010. Previously, it was replaced with the term of impaired (Ban điều phối các hoạt động hỗ trợ người tàn tật Việt Nam 2010). Almost CWD in interviews expressed their expectation on being called with “people with disabilities” rather than with the name of disability types. Being sad and feel frustrated in case of being disabled as the common feelings in which CWD and their family expressed. However, almost CWD and their parents are very positive on their situations and try to overcome their difficulties by themselves.

On looking back to responds to the KAP of disability and implications from the interviews and observations, almost responds favour more towards positive feedbacks about these definitions’ contents. The social construction of disability is discussed with contents relating to responses about their KAP (knowledge, attitudes and practice toward disability definition and the social status of PWD).

Findings from survey with 210 respondents from PWD and PWND’s voice are confirmed that the general understandings on disability are still limited:

- The understanding on disability is mainly based on medical/individual model that focuses on the disability’s causes in words of health or individual problem rather than viewing the social causes in aspects of the social barriers and restriction.
- Social attitude toward disability and PWD seems to be very empathetic, however it is less regarded to CWD’s ability as well as there are more attitudes on charity giving and supporting than helping them to be independent in their life.
- And finally, in the practice aspect, there are more positive responds in the statement of “being along with CWD/PWD” rather than in one of “being close friend with them”.

These findings are compatible with other research findings in Vietnam recently (UNICEF Vietnam 2010; Duong et al. 2008; UNICEF, MOLISA MOLISA 2011). These research focuses on the situation of PWD and their life, almost taken from the voice of PWND and they also consider more on inclusive education, economic contribution of PWD and rehabilitation services for PWD as well.

In additions, the further discussions around physical limitations and the situations of social policies on disability are subordinated as the other approaches. Such limitations are stated in terms of the voices of both PWD and PWND. While PWND express their lack of understanding on disability, PWD demonstrate more positive awareness but they tend to be self-discriminated. These situations also make the problem of social inclusion of PWD in general and CWD in particular more severe.

The way in which disability is constructed socially based on personal difficulties and medical conditions is more preferable in this study rather than considers to the social barriers. The latter aspect is stated clearly but almost in term of the facilities for inclusive education, travelling or having recreational activities. However there is lack of wishes and expectations for requirements done by social sides while reminding the expresses for social changes.

The understandings on disability have been more specific and suitable with the contexts, in spite of the existed ideas on the individual model or medical model on disability. The changes from individual model to social model on disability have been taking long time as in the developed and Western countries, it is also required to make the change on the social movements on supporting PWD, on changing social and physical conditions for PWD as well as making any changes on social awareness on disability by PWD and PWND concurrently instead of by PWD or PWND.

Disability, in Vietnam, has been largely used in academic and non-academic paper since the approval of Law on People with Disability (2010) however in daily life it is witnessed the discrimination toward PWD as well as CWD in term of travelling, inclusive education, recreational activities. There is big gap on social awareness on disability in theory, legal documents and practices. As recommended by NGOs and organizations for PWD, it needs having more and long-term strategies on dealing with this situation. Promoting the social awareness on disabilities requires the systematic approaches in social policies, communication and in practices as well as in community development approaches which participated with families of PWD, PWD, and societal organisations in national wide.

The changes for understanding and constructing the meanings of disability are currently on the continuity from individual model to social model.

### Dealing with difficulties: voice of CWD

CWD did not blame their difficulties caused by the limitation on inaccessible buildings. They always found these difficulties caused by their own and they deal with them by adapting rather than requiring the changes from society. They expressed and showed their interactions basing the practical conditions and from other CWD’s interactions in their contexts*.* Such interactions and their daily activities are meaningful for them, in their mind and their daily life. Statements about the difficulties in aspects of un-accessible buildings, limited spaces for playing are mostly expressed by CWD when talking about the difficulties in their daily life.

The other ways to deal with these difficulties at school are by self-help group that is powerful than supports from other CWND. Almost CWD, in situation of vision disabilities, were trained with rehabilitation skills and involved in self-help groups. They try to make their strengths from their groups by learning the life skill together. CWD is closed in asking for their supports from other CWND and teachers in their study. They prefer to ask their peer friends in residential areas rather than from CWND in classroom. As their awareness, asking for help from those in the same conditions and situations is easy and convenient than from other PWND. It is also found that CWD also provide their ideas of supports from PWD, which are given with more sympathetic than from PWND. In this aspect, social awareness to ward disability and PWD in general and CWD in particular is still limited from both PWD and PWND. This awareness also increases the preferred trends on institutionalisation of education and social support for PWD and CWD.

### Factors for social inclusion in schools

From the survey and interviews, teachers and peer students, social awareness on disability are significant factors for social inclusion of CWD in schools settings. Such findings are similar with other implications from recent research in Vietnam. Other ideas about the social workers in schools, social charities are not highly recommended. The main reasons for such problem are that social work is a new professional area in Vietnam and has not been applied directly as a current social service in schools, so social work is still hidden from the voice of PWD and PWND.

Teachers are considered as the main factor for improving social inclusion process for CWD in school, they are critical for directing any supports from CWND toward CWD in learning, playing and making connections between them. Teacher is so powerful in the view of children in school, that’s a traditional value in Vietnamese society.

## Conclusion

This paper provided the results of this research which examined experiences of CWD in school in Vietnamese contexts throughout their disadvantages and advantages in schools, ways to dealing with these disadvantages by self-adaptation, self-help, and to be active in initiating all social relationships with CWND, teachers and support workers. Disadvantages and difficulties expressed by CWD are the contents of limitations on learning facilities, which are more prominent than those on social attitudes, social policies and social supports. Those on mobility and social supports are also acknowledged but CWD always think that their reality is quite good and they are happy with that. They are aware that they are happier than those CWD who are unable to attending schools. Almost difficulties, CWD are facing in daily life at schools, are socially constructed. The ways to dealing with difficulties in school are exposed and they are originated from self-adaptation and self-determination of CWD in their life. In order to overcome their situation and to have chance to contribute society and to become independent in their life, CWD consider to follow the education, to have vocational training are best ways for them. Teacher and CWND in school are key factor for improving the social inclusion process for CWD as well as those supporting CWD on deal with the difficulties in schools.

It is recommended to promote any activities on increasing social awareness on disability and the social status of CWD. In which CWD are able to attend, to make their voice and to contribute to. School setting is the most significant one for CWD to make any changes on their social awareness and on accessing the sustainable inclusion process.

## Author’s information

Tran Van Kham, finished his PhD in Social Work and Social Policy, from University of South Australia in 2012. He is currently working as Deputy-Director of Office for Research Affairs, Hanoi University of Social Sciences and Humanities-Vietnam. His recent published papers and his main research are on social work, community development, disability, social inclusion and community cohesion. Correspondence to Tran Van Kham at: khamtv@ussh.edu.vn.
